# Assessing the Effect of Dynamic Capabilities on the ESG Reporting and Corporate Performance Relationship With Topic Modeling: Evidence From Global Companies

**DOI:** 10.3389/fpsyg.2022.898935

**Published:** 2022-05-11

**Authors:** Byung Mo Yang, Oh Suk Yang

**Affiliations:** Department of Business Administration, Kangwon National University, Chuncheon, South Korea

**Keywords:** dynamic capabilities, ESG reporting, topic modeling, organizational adaptation, uncertainty, corporate performance, Word2Vec embeddings

## Abstract

The primary purpose of this study is to examine the relationship between the dynamic capabilities (DCs) embedded in ESG management, which are being pursued by global companies, and corporate performance amid increasing uncertainty. Furthermore, the secondary purpose is to examine the function of environmental uncertainty moderating the DCs-performance relationship. Concerning the analysis tool, this study employs topic modeling with Word2Vec embedding that analyzes unstructured data. This was employed as an alternative method beyond the limitations of the traditional approach, i.e., survey or interview. A DCs dictionary was constructed by redesigning the 12 detailed dimensions of Teece’s DCs into 10 dimensions, and then time series scores of individual global companies were extracted by applying this dictionary to the sustainability reports of 97 companies. Sustainability reports of 153 companies among Fortune Global 500 companies announced in 2020 were originally collected, but in the process of collecting additional financial data about these companies from OSIRIS, a total of only 97 companies was selected in the end due to omission of data. A fixed effect panel analysis was conducted, and the main findings are as follows: First, the DCs embedded in ESG management have a positive or negative direct effect on corporate performance. In particular, a statistically significant relationship was not observed in the innovation (technology) oriented capabilities, whereas a statistically significant positive relationship was observed in the customer (market) oriented capabilities. Second, uncertainty moderates the relationship between DCs and corporate performance positively or negatively. Interestingly, the moderating effect of uncertainty only appears in the function of the sensing and reconfiguring capabilities. From this, it can be seen that the function of DCs, which is embedded in the ESG management of global companies, is limited due to the imbalance between the sensing-seizing-reconfiguring capabilities. These findings imply that, despite the positive function of DCs embedded in ESG management, costs and benefits occur at the same time, and DCs can improve performance only if there is an organizational adaptation strategy suitable for uncertainty. Accordingly, business managers need to recognize the importance of pursuing sensing-seizing-reconfiguring capabilities in a balanced way to improve corporate performance through ESG management under uncertainty.

## Introduction

After the era of emphasizing Corporate Social Responsibility (CSR) and Creating Shared Value (CSV), global companies are currently confronting a new topic of ESG (Environment, Social, Governance) management. As technological and market changes accelerated, the concept of values demanded by society from companies diversified, expanding the scope of corporate citizenship, and now it has begun to emphasize socially responsible investment to investors (including companies). In line with changes in the corporate business environment, global companies today publish annual sustainability reports, attempt to shift the paradigm for ESG management beyond the concepts of CSR and CSV, and form a new strategic architecture to secure competitive advantages. An increasing number of companies are publishing sustainability reports for the purpose of sharing their socially responsible management performance with stakeholders. This is because it is an integrated information disclosure tool and at the same time can highlight the image of communicating with society. Therefore, it is worth noting whether this new strategic architecture can secure competitive advantages beyond the scope of CSR or CSV.

From a traditional Resource-based View (RBV), ownership-specific competitive advantages associated with firm-specific attributes are emphasized as performance determinants. The logic that resources determine performance makes companies focus on strategies that retain sustainable competitive advantages. The problem is that the sustainability of competitive advantages is likely to be threatened by market dynamism. Market dynamism frequently changes the value of key factors for competitive advantages, shortens the period of maintaining a company’s sustainable competitive advantages over time, and creates companies that recover again after losing their competitive advantages (Wiggins and Ruefli, 2002). Thus, under uncertainty, companies tend to develop temporary competitive advantages over sustainable competitive advantages, and these temporary competitive advantages are linked like a chain, allowing them to maintain high performance over a long period of time (Wiggins and Ruefli, 2002).

Research, on the other hand, from the Dynamic Capability View (DCV) denies the relationship that resources determine performance, and explains that dynamic abilities mediate or moderate the relationship between resources and performance. Wu (2006), analyzing 244 Taiwanese IT companies, argues that resources such as special know-how, financial capital, sales management capability, reputation, and partnership experience do not directly affect corporate performance, but improve performance in areas such as innovation speed, market response speed, production efficiency and flexibility, and R&D capability through dynamic capabilities (DCs).

Current research on the DCs that are emphasized as companies’ alternative strategic assets in an era of uncertainty examines the DCs’ effects in certain partnership modes such as strategic alliances and joint ventures (Cohen and Levinthal, 1990; Doz and Hamel, 1998; Madhok and Osegowitsch, 2000), their relationships with competitive advantages such as product quality, process flexibility, and low cost (cost effectiveness; O’Reilly<suffix>III</suffix>, and Tushman, 2008; Li and Liu, 2014; Vanpoucke et al., 2014; Kuo and Tsou, 2017; Yu et al., 2017), their influence on corporate performance (Helfat, 1997), their relationships with environmental dynamism (Li and Liu, 2014), and their functions to create new competitive advantages (Helfat, 1997). It is noteworthy that all of them are based on static approaches. Previous studies on the moderating and mediating effect of DCs on the competitive advantages-corporate performance relationship (Wu, 2006; Jiao et al., 2019) also explore the interaction between firm-specific advantages and DCs, but since they also employ static approaches, they are not free from critique either (Collis, 1994; Winter, 2003; Schilke, 2014).

Unlike environmental determinism, which describes the appearance of organizations responding to changes in the business environment, organizational adaptation to environmental uncertainty shows constructivism consisting of interactions between companies and the challenges to the business environment. From the perspective of organizational adaptation to environmental uncertainty, it is difficult to fully understand DCs of current significance embedded in ESG management strategies using traditional static approaches, so a new alternative analysis tool is needed.

This study, therefore, aims to empirically examine the direct effect of DCs on the ESG reporting and corporate performance relationship and the moderating effect of uncertainty on the DCs-performance relationship by opening a black box of DCs, which are evaluated as one of the influential theoretical lenses in the academy of strategic management (Kim and Heo, 2016). It is also intended to empirically examine how companies respond to and proactively approach new technologies and market changes. Concerning a specific research methodology for these research purposes, based on Teece’s (2007) dynamic capability concept, a dynamic capability dictionary has been constructed using topic modeling with Word2Vec embedding, and applied to the empirical analysis of a company’s strategic architecture for an ESG management.

The remainder of this study is organized as follows. The above Introduction presented the main research questions addressed by this study. Next, the Literature Review and Hypotheses section not only explains the theoretical concepts and analysis tools used in this study, but also presents the hypotheses that this article intends to review. The next section, Research Methodology describes model specification, such as the research model, variables, and measures. Empirical Results summarizes the analysis results, and the Discussion section describes the theoretical and managerial implications derived from the analysis results. Finally, the Conclusion presents the methodological limitations of this study and future research themes that were not addressed in this study.

## Literature Review and Hypotheses

### ESG

The ideological roots of the ESG concept stemmed from the concept of “social capital” presented by James S. Coleman, 1988 as a new concept for value measurement against Milton Friedman’s theory that corporate evaluation in the 1960s and 1970s should be based on net profit. Since then, in a book by Elkington and Fennell (1998), social, environmental, and economic factors have been emphasized as the determinants of the corporate stock value. Putnam’s (1993) concept of social capital consisting of moral obligations and norms, social values (especially trust) and social networks (especially voluntary associations) also forms another root of ESG values.

An official interest in ESG began in 2000 when the European International Organization Global Reporting Initiative (GRI) presented guidelines for the preparation of sustainability reports for companies, presenting standards including more than 150 indicators covering the ESG sector. Thereafter, the ESG concept was used in the 2003 UN Environmental Planning Finance Initiative, and the ESG concept was officially used in the 2005 UN Global Compact. In addition, the Financial Stability Committee, established in 2017 with the delegation of the G20 and the central bank, recommended disclosure of financial information on climate change. Through this series of formal procedures, companies are obligated to identify climate change-related risks and opportunities, reflect them in risk management systems and strategies, quantify the expected financial impact, and post them.

Comparing the concept to CSR (Carroll, 1999; Lin et al., 2022), which emphasizes corporate citizenship based on philanthropic responsibility as a social member, CSV has since embodied CSR into corporate management and formed a strategic architecture that creates shared value between companies and society (Kramer and Porter, 2011). The LVMH’s Mécénat initiative to sponsor the restoration of a World Heritage Site, Marriott Hotel’s project to support the poor, and Toyota’s forest protection activities are understood as CSR categories. Meanwhile, the Matsushita Electric’s investment in developing non-Freon refrigerators, the development of no margin meningitis vaccines by Glaxo Smith Kline, and UPS’s safe driving training for teenagers are classified into CSV categories.

ESG management refers to challenging environmental impact (E), social impact (S; workers’ health and safety, and diversity), and governance (G; corporate ethics, shareholders’ rights, and executive compensation policies) by using non-financial factors such as energy and materials. This is the case in which SK Hynix in Korea was the first among the world’s memory semiconductor companies to issue a US$1 billion bond for eco-friendly business investment under the strategic architecture of focusing on the development of a new state-of-the-art wastewater treatment plant, water recycling system, and low-power SSD. Another example of ESG management is that in 2016, Danish startup ‘Too Good To Go’ operated a restaurant closing discount platform that sells leftover food at buffet restaurants at a certain time. The case of Patagonia, an outdoor brand where 60% of the jackets on sale are made of recycled materials, is also a good example of ESG management.

### Dynamic Capabilities

Despite the existence of conceptual differences between researchers (Williamson, 1999; Priem and Butler, 2001), DC is largely classified into two competing viewpoints such as the RBV and the Capability-based View (CBV). In the former, DC is conceptualized as a routine or process that integrates and reorganizes resources or resource bases (Eisenhardt, 1989; Eisenhardt and Martin, 2000), or as a pattern of collective activity (Zollo and Winter, 2002) that creates or modifies operational routines from a broad RBV that recognizes DC as another type of resource as the ability to create resources.

In the case of CBV, DC is also recognized as the ability to create resources, similar to RBV, and it is conceptualized as the ability to integrate, build, and reconfigure capacities or routines created by resources (Teece and Pisano, 1994; Teece et al., 1997; Teece, 2007). Alternatively, it also may refer to the ability to change the company’s routine, activity, and the process itself which results in changing the resource base (Collis, 1994). Therefore, DC is a parent concept of the black box that challenges the routines, activities, and processes in which resource conversion occurs.

In this study, according to the conceptualization of Teece (2007), DC is defined as sensing-seizing-reconfiguring capacities. Sensing capabilities refer to analytical systems and individual capabilities to learn, detect, filter, shape, and measure technological and market opportunities. Seizing capabilities refer to corporate structure, procedures, design, and incentives to occupy technology and market opportunities. Reconfiguring capabilities refer to the corporate ability to continuously (re)align firm-specific tangible and intangible assets and operating modes in line with market changes. The absorptive capacity to evaluate and acquire external information or knowledge (Cohen and Levinthal, 1990) is in line with the seizing capabilities, while the combinative capability to realize technology or market opportunities with outcomes (Kogut and Zander, 1992) by combining and utilizing existing knowledge is similar to the reconfiguring capabilities. In addition, the learning ability to improve existing operational capabilities with new knowledge, and the coordination ability to coordinate or deploy resources and activities (Pavlou and El Sawy, 2011; Nieves and Haller, 2014; Vanpoucke et al., 2014) also compose Teece’s (2007) DC in a great detail.

DC consists of sensing-seizing-reconfiguring capabilities, and each capability is further subdivided into four activities, respectively (Teece, 2007). First of all, sensing capabilities consist of four detailed activity areas: (1) internal R&D and selection processes of new technologies, (2) monitoring processes of external science and technology development, (3) monitoring processes of innovation within the supply chain and target market segments, and (4) observation processes of changes in customer needs. Seizing capabilities consist of four detailed activities: business model design, selecting corporate boundaries, selecting investment decision protocols, and building organizational loyalty and commitment (organizational efficiency management). Finally, reconfiguring capabilities consist of four detailed activities: decentralization, co-specialization, governance, and knowledge management (Teece, 2007). Today, companies publish sustainability reports responding to the uncertainty caused by the market needs that emphasize ESG management, and display their DCs to sense changes in the business environment, to seize new market opportunities by changing business models, and to reconfigure resources and capabilities.

### Hypotheses Development

#### Strategic Postures of Dynamic Capabilities to Achieve Corporate Sustainability

##### Sensing

In the study of Teece (2007), sensing consists of a process to direct internal R&D and select new technologies, a process to tap developments in exogenous science and technology, a process to tap supplier and complementor innovation, and a process to identify target market segments, changing customer needs, and customer innovation. The four sessions of sensing activity (capability) can be classified into two subsectors: the Analyzing capability to analyze the environment of external innovation, internal innovation, and R&D activities to learn market opportunities; and the identifying capability to recognize market segments, ecosystems and industrial trends, and changing customer needs. The former can be summarized as innovation (technology) orientation, and the latter can be summarized as customer (market) orientation. In the same vein, Ordanini and Parasuraman (2011) classified DCs into customer-oriented DCs and innovation-oriented DCs.

Technology orientation is achieved by coordinating the structure, system, and resources of a company in line with technological changes and using this technology as an organizational competency. Customer orientation refers to the alignment of organizational resources to create an excellent customer value. The problem is that if a company adopts only innovation orientation for technological change and overlooks customer orientation factors, it cannot meet consumer needs (Ordanini and Parasuraman, 2011). It is necessary to create joint value with customers and innovate products and services by combining customer orientation and utilizing corporate resources (Lusch et al., 2007). Using only new knowledge and resources to pursue innovation cannot guarantee consumer satisfaction. Therefore, based on changes in organizational systems and strategies that have shifted to meet technological changes, companies should adjust their organizational capabilities and innovation capabilities to suit customer needs to actively cope with environmental changes. And all innovation activities can create positive customer perception and consumer demand only when they are based on customer-oriented DCs. Customer-oriented companies can develop and maintain close relationships with customers and get quick feedback from customers (Shapiro, 1988; Fundin and Bergman, 2003). If a company does not actively lead customer orientation, competitors will preempt consumers’ unmet demand (Paladino, 2007). In response to the aforementioned reasoning, the following hypotheses are assumed:

*Hypothesis 1: Analyzing capability of dynamic capabilities will have a positive influence on corporate performance in seeking an ESG strategy.*

*Hypothesis 2: Identifying capability of dynamic capabilities will have a positive influence on corporate performance in seeking an ESG strategy.*

##### Seizing

According to Teece’s (2007) argument, as the market and technology change, the capability to seize the newly emerging market opportunities results in a company’s competitive advantages. A company’s seizing capability requires the relevant corporate structure and procedures, business model design and incentive systems (Teece, 2007). A company’s seizing activity for market opportunities begins with the development and investment for commercialization, and redesigning of their business model. Based on fundamental strategies (Porter and Kramer, 1985), such as cost leadership or differentiation, a business model design that reflects organizational architecture for productivity and efficiency and customer architecture for target customers should be executed (Teece, 2007).

In the newly designed business model, the timing of resource commitment, commercialization strategies, and investment priorities to seize market opportunities are defined (Teece, 2007), and enterprise boundaries are set according to technological changes. Enterprise boundaries should proceed so that the benefits of innovation can be greater than those of imitation, and should be able to establish a cospecialization strategy to prevent the occurrence of cannibalization in which new products erode old product marketing (Teece, 2007).

As such, business model design and enterprise boundaries resetting according to technological and market changes are bound to require reorganization of the organizational decision-making protocols. Companies attempting to establish new strategies in response to environmental uncertainty should choose effective decision-making protocols so as to avoid decision-making errors that do not take into account strategic inflection points which often determine a company’s survival and prosperity (Teece, 2007).

Global companies’ seizing activities (capabilities) in response to uncertainty also require changes in organizational culture. Companies’ active organizational adaptation to environmental changes requires efficient communication and leadership based on entrepreneurship, which consists of proactiveness, innovation, risk-taking, and agility (Drucker, 1985; Covin and Slevin, 1991; Kao, 1993; Gartner et al., 1994; Wooldridge and Jennings, 1994; Lumpkin and Dess, 1996; Davis and Harveston, 1998) and an effective communication system (Pincus, 1986; Ford and Ford, 1995). The sharing of corporate vision is also important (Robbins and Duncan, 1988; Kotter, 1990; Evans and Doz, 1999; Teece, 2007). Corporate shared vision is often considered in line with leadership (Finkelstein et al., 1996; Conger and Kanungo, 1998; Wang et al., 2011). This sequence of organizational culture and system transformation can properly develop a business environment, and in turn, it plays an important role in developing a successful organization and developing trust and commitment within the organization (Kotler and Keller, 2006). Here, “commitment” refers to the job commitment and organizational commitment that constitute organizational efficiency, which is said to play a role in triggering the improvement of innovation and financial performance (Campbell, 1977; Hall, 2002).

In terms of organizational efficiency, the DCs to build company’s competitive advantages are expected to have a positive correlation with corporate performance. In addition, the positive correlation between loyalty and commitment building and corporate performance has the potential to improve the reconfiguration and deployment of organizational resources. Therefore, the positive DCs to seize market opportunities due to technological and market changes, the efficient communication structure, and the possession of an innovative organizational culture are key sources of corporate competitive advantages. To sum up, regarding the detailed seizing activities (capabilities) of DCs the following hypotheses are assumed:

*Hypothesis 3: Delineating business model of dynamic capabilities will have a positive influence on corporate performance in seeking an ESG strategy.*

*Hypothesis 4: Selecting enterprise boundaries of dynamic capabilities will have a positive influence on corporate performance in seeking an ESG strategy.*

*Hypothesis 5: Selecting decision-making protocols of dynamic capabilities will have a positive influence on corporate performance in seeking an ESG strategy.*

*Hypothesis 6: Building loyalty and the commitment of dynamic capabilities will have a positive influence on corporate performance in seeking an ESG strategy.*

##### Reconfiguring

From the DC perspective, theorists stress that the key to sustainable profitability is the ability to recombine and reconstruct assets and organizational structures as companies grow and markets and technologies change (Schilke, 2014; Girod and Whittington, 2017). Companies must continuously align and realign internal tangible and intangible assets to suit environmental changes for sustainable growth (Teece, 2007). The continuous alignment and realignment muse be associated with decentralization and cospecialization (Teece, 2007). In addition, changes in the organizational structure are also required, and thus governance should be changed to lower costs derived from the agency problem. Also, organizational learning skills should be cultivated for efficient knowledge transfer and know-how integration (Teece, 2007).

Intellectual assets within the organization should be integrated so that they can adapt to technological changes and further lead technology development, and insufficient intellectual assets should be absorbed through open innovation. Open innovation refers to innovation in which knowledge resources such as valuable ideas, know-how, and physical technology are commercialized from within or outside the company (Chesbrough, 2006). Therefore, it is necessary to resolve innovation resistance within the company. This type of resistance may lead to destructive knowledge creation through knowledge transfer and knowledge sharing associated with integrated mechanisms. Meanwhile, it is also necessary to strengthen organizational learning of technology and knowledge resources outside the company. During this process, companies need to boldly promote either open innovation through strategic alliances, or joint ventures for organizational growth while they coordinate, reconfigure, and recombine technologies and assets (Teece, 2007).

Regarding open innovation, one thing to note is the strategic fit. In response to changes in technology and customer needs, companies need to invest in short-term product development and long-term research activities, i.e., exploitation and exploration (March, 1991), and look for co-specialization between new and old products (Teece, 2007). Considering the learning race that may occur, even between companies, it is also necessary to carefully consider differences in knowledge level and content/knowledge specialization.

Corporate strategy transformation according to environmental changes, on the other hand, requires an organizational redesign. In order to attract modern talent, the incentive system should be strengthened. In addition, to establish an effective management of open innovation and to protect intellectual assets, potential conflicts in joint R&D activities should be resolved, eliminating costs from the agency problem. Such governance improvement capabilities prevent rent-seeking activities between partners in advance and enable knowledge transfer and knowledge sharing to proceed in a proper way.

Companies should be able to achieve effective knowledge transfer not only inside the company but also outside (other companies or overseas subsidiaries) in the process of reorganizing knowledge management due to environmental changes. Effective knowledge transfer creates knowledge sharing, a major process of knowledge management activities (Kogut and Zander, 1992), and this knowledge transfer and knowledge sharing are fundamental means for members of the organization to contribute to knowledge application, innovation, and ultimately competitive advantages (Jackson et al., 2006).

According to the current business environment elements emphasizing ESG management, global companies improve sustainable R&D and knowledge management capabilities through the reconfiguring capabilities of DCs as a special resource embodied within the company (Eisenhardt and Martin, 2000; Makadok, 2001). They also align heterogeneous organizational resources to environmental changes, and establish corporate governance to begin and improve cospecialization, resulting in better performance. Considering the positive function of reconfiguring activities, this study assumes the following series of hypotheses:

*Hypothesis 7: Cospecialization of dynamic capabilities will have a positive influence on corporate performance in seeking an ESG strategy.*

*Hypothesis 8: Governance of dynamic capabilities will have a positive influence on corporate performance in seeking an ESG strategy.*

*Hypothesis 9: Knowledge management of dynamic capabilities will have a positive influence on corporate performance in seeking an ESG strategy.*

*Hypothesis 10: Decentralization of dynamic capabilities will have a positive influence on corporate performance in seeking an ESG strategy.*

### The Moderating Effects of Uncertainty on the Dynamic Capabilities- Performance Relationship

The business environment is an important factor for companies to consider in the process of acquiring necessary resources and establishing strategies to achieve competitive advantages (Achrol et al., 1983). Therefore, changes in the business environment are management resources that companies must continuously monitor. Environmental uncertainty as a management resource imposes opportunities or threats on individual companies. Certain companies get market opportunities from technological and market changes, while certain companies face threats. So far among the previous studies, there is no consensus about the effect of environmental uncertainty on corporate performance.

As technological changes accelerate and customer needs diversify, companies’ strategic goals face continuous challenges. Technological changes lead to costs by imposing restrictions on the use of scarce resources and capabilities of companies. However, looking at the company’s response strategy, as uncertainty increases, companies try to strengthen their internal capabilities, access high-quality information for innovation, and solve problems through a strategic move to expand networks that actively utilize external resources (Uzzi, 1997; Koka and Prescott, 2002).

It is still difficult today to conclude that major conglomerates are making strategic moves in ESG management. Even the function of firm size, which controls the positive relationship between ESG management and corporate performance, does not show consistency. In addition, understanding of the relationship between ESG management and corporate performance is also divided into negative and positive viewpoints. Nevertheless, as can be seen in many corporate cases, the organizational adaptation shown by global companies in response to the increase in uncertainty brought about by environmental dynamism of the importance of ESG management leads us in part to expect uncertainty to have a positive effect on corporate performance.

In summary, unlike the negative view that increasing uncertainty due to changes in the business environment imposes constraints on companies, it can act as an incentive to increase internal capabilities and shift the paradigm of competitive advantages to realize corporate problem solving and improve performance. In this study, from a neutral standpoint on the role of environmental uncertainty, we intend to examine the relationship in which environmental uncertainty moderates the DCs of global companies embedded in ESG management strategies. Specifically, the positive and negative functions of uncertainty are expected to be inconsistent when interacting with the detailed sub-activities (capabilities) of DCs, and differ depending on the sensing-seizing-reconfiguring capabilities. This is because when individual companies pursue organizational adaptation strategies based on ESG management in response to environmental uncertainty, they show an imbalanced pattern of implementation between sensing-seizing-reconfiguring capabilities. Accordingly, the following hypotheses are assumed:

*Hypothesis 11: Environmental Uncertainty will moderate the Dynamic Capabilities-Corporate Performance Relationship.*

## Research Methodology

### Text Mining: Unsupervised Learning of Latent Dirichlet Allocation

Supervised learning is a method of learning data using data with correct answers, and unsupervised learning is a method of predicting results for new data by clustering data without correct answers among similar attributes. A classification model or regression analysis belong to the former, while clustering analysis is a good example of the latter. Text Mining, which derives meaning from a large amount of text data, is also one of the unsupervised learning methods. Text is not structured data such as general numerical data, but unstructured data. The relationship or mode, i.e., pattern, between and among data is embedded in the unstructured text. Text mining is a machine learning technique that examines connections between various diverse sources of information and derives relationships or patterns, beyond the benefits provided by a simple content analysis.

Topic modeling is one of the main methodologies constituting text mining techniques. Topic modeling infers story topics by finding clues contained in the context and grouping words with similar meanings (clustering). In this process, stochastic techniques are used to discover hidden semantic structures, and the work is divided into several stages. The first step is to collect text data from relevant data (all forms of text such as reports, newspaper articles, broadcasting news, and social media). Subsequently, the second step extracts the most frequently used and key words from the preprocessed data. The most frequently used words are identified by arranging the words in the order of high word frequency throughout the total document. Key words are extracted using various weighting methods such as Okapi BM25 and Term Frequency-Inverted Document Frequency (TF-IDF). In particular, the TF-IDF weighting scheme identifies how important words are within a particular document and then sorts them using the figures from them to consider the highest-valued words as key words (Santhanakumar and Columbus, 2015; Dai, 2018; Qaiser and Ali, 2018).

The next third step can create and analyze a semantic network that can identify the relationship between each word based on the extracted word. It is a method of linking words that appear together for each article and expressing them as an adjacency matrix. Thereafter, related topics are created and analyzed through the topic modeling package. Topic modeling is one of the research methods used in text mining, and is a process of finding topics in a set of documents. In detail, there are Latent Semantic Indexing (LSI), Deerwester et al. (1990), pLSA (Probabilistic Latent Semantic Analysis; Hofmann, 1999), and LDA (Latent Dirichlet Allocation; Blei et al., 2003), and among them, this study employs the LDA method.

This is because the LDA method is recent enough to complement the shortcomings of the earlier methods (Blei, 2012). The LDA scheme assumes that documents consist of a mixture of topics, and topics form a dirichlet distribution, which is one of continuous probability distributions, not a binomial distribution. The sum of the topic words is 1, which is the same as all of the topic element values (Blei et al., 2003). LDA is an unsupervised learning algorithm that generates topics by estimating the topic distribution of each document and the word distribution within each topic, representing the probability distribution in which documents and words are assigned to the topic. Subsequently, in step 4, the generated topic network is analyzed. It is a method of numerically presenting the structure by modeling the relationship between topics as a node and a link. Through this, words related to a specific topic and topics being discussed can be identified, and the correlation between and among topics can be analyzed.

### Building Lexical Dictionary of Dynamic Capabilities Employing Word2Vec Embedding

It is possible to form a word dictionary through similar contextual analysis by employing specialized words with semantic similarities. In particular, technologies such as Word2Vec using machine learning are able to construct vector space in a similar context for text corpus of natural language. By using cosine similarity function, a distance between vectors close to each other in this vector space has the property of revealing words that are semantically similar or related to a given word. That is, when the machine learning method is used, each word is expressed as a correlation between similar meanings and other words. This unstructured text analysis has been revised to improve the rigor of the correlation between and among words (Mikolov et al., 2013).

After the introduction of Word2Vec by Mikolov et al. (2013), in order to discover the characteristic vocabulary of the professional dictionary, it is numerically determined whether words are semantically related in the dictionary. In this study, an algorithm was designed to construct a given word dictionary. Using this algorithm, from this corpus, it is possible to extract characteristic vocabularies for domains and build more complex lexical structures, which taxonomy employs. The corpus we explored for building DCs dictionary comes from a paper by Teece (2007) and contains the following text: 507 words were extracted based on centrality and linkage in the sensing dictionary, and 138 keywords were finally extracted. As a result of a heuristic review of 587 words, 137 keywords were extracted for the seizing dictionary, and 134 keywords were extracted for the final reconfiguring dictionary. In the case of seizing, four keywords [loyal, culture, affordability, regeneration (s)] were added through the heuristic approach and eight keywords such as fitness, fit, value, knowledge, know-how, open, and embraced were added for reconfiguring dictionary as shown in Table 1.

**TABLE 1:** Extract key words for each DC dictionary.

Dynamic capability	1st Step Number of Extract key word centrality cut off	2nd Step Heuristic	After Heuristic additional key word
Sensing	507 words	Decreased to 138 words	Not applicable
Seizing	587 words	Decreased to 137 words	Loyalty, culture, appropriability, revenue
Reconfiguring	565 words	Decreased to 134 words	Fitness, fit, value, knowledge, transfer, know-how, open, embraced

### Word2Vec Word Embedding to Vector

In this study, Word2Vec (word embedding to vector) is used as a method of measuring the networking similarity between words developed by Google in 2013. This method has a stochastic language model using a single-layer artificial neural network technique (Mikolov et al., 2013). Stochastic Language Model learns context through words and expresses the meaning of words.

Word2Vec is applied to the vector space model of the query document with centrality and similarity value. The vector space representation of the words provides a projection of words from the documents with similar meanings. It is a learning algorithm based on the distributional hypothesis of linguistics (Harris, 1954; Sahlgren, 2008; Le and Mikolov, 2014) that words with the same meaning often appear in similar contexts. As learning progresses, words with similar meanings have similar vector positions. It is taught in a way that maximizes the probability of a word that can be inferred from a specific context, and after learning, similar words have similar vector positions. The similarity also increases. In the end, the similarity between words is measured to be higher as they are closer in context.

### Model Specification

#### Sample and Data Collection

The method of collecting previous reports to calculate the quantitative index value of DCs is as follows. Companies selected as the Fortune Global 500 in 2020 provide ESG reports as PDF files in the corporate filings archives, and anyone can access and download files. In this study, data for a panel model over 10 years was collected by securing annual ESG reports from a total of 153 companies from 2011 to 2020. In order to analyze the collected ESG management reports, data conversion was performed to analyze unstructured data by converting PDF files into TXT files. Next, the score for each activity is obtained by performing a similarity analysis between the codebook and the sustainability management reports that have been pre-built DCs.

Financial data is secondary data that shows major financial attributes collected from the OSIRIS database. OSIRIS databases provide useful information such as firm-specific variables, financial profitability, activity, stability, growth, and market performance. Finally, the data configuration for a total of 97 companies was completed since the data value for financial variables to be put into the research model was omitted or not secured. The major industrial sector consists of manufacturing and service companies such as agriculture, mining, science and technology, construction, wholesale and retail, transportation and warehouses, bio, chemistry, engineering, health, IT, and automobile.

#### Variables and Measures

The variables put into the empirical analysis of this study are variables related to designed DCs score, environmental uncertainty, and financial performance. The score of DCs represents the activity level of the dynamic capacities inherent in the ESG reporting. If the score is high, it can be said that the corresponding activity is actively progressing. Conversely, if the score of competency activity is low, *vice versa*. The research method used in other studies through literature review was used as a starting point, and in particular, the variable measurement is presented in Table 2.

**TABLE 2:** Variables and measures.

Variables	Acronyms	Measures
ROA	ROA	Return on asset = Net profit before taxes/total assets
Tobin’s Q	TBQ	Ratio of a company’s market value to total assets
Analyzing	ANL	External and internal innovation/open innovation focused/External and internal R&D
Identifying	IDN	Market segments/changing customer needs/valuating ecosystem and industry trends/sense opportunities and threats
Enterprise boundaries	EBR	Arranging alliances to upgrade/deciding outsourcing and insourcing/controlling bottleneck assets/assessing legal and natural protection through an appropriability regime
BM design	BMD	Selecting technology/feature and product/service architecture/(Re-)Designing revenue structures/(Re-)Designing cost structures/Designing mechanisms to capture value/Designing partnerships
Decision-making protocols	DMP	Recognizing inflexion points/avoiding and mitigating decision errors/avoiding anticannibalization tendencies/encouraging creative thinking and action/encouraging removal of no value-adding assets and activities/learning from mistakes
Loyalty and commitment	LYC	Demonstrating leadership/communicating effectively/recognizing non-economic factors, value and culture
Cospecialization	COS	Managing strategic fit so that asset combinations are value-enhancing
Governance	GOV	Achieving incentive alignment/minimizing agency issues/checking strategic malfeasance/blocking rent dissipation
Knowledge management	KNW	Learning/transferring knowledge/integrating know-how/achieving know-how
Decentralization	DEC	Developing integration/coordination and reconfiguration skills/adopting loosely coupled structures/embracing open innovation
Uncertainty	UNC	A rate of change in sales profit and the standard deviation of the analysis is calculated as an environmental volatility value
R&D intensity	RND	R&D intensity = R&D expenditure/operating revenue (%)
Price Earnings Ratio	PER	Market value per share/earnings per share
Revenue per employee	RPE	Productivity = operating revenue/no. of employee (logarithm)
Current ratio	CR	Current assets/current liabilities (%)
Debt ratio	DB	Total liabilities/total assets (%)
Firm size	FS	Total assets (logarithm)
Firm age	Number of years since first date of incorporation	

In this study, the quantitative score of DCs is a measure of sensing, seizing, and reconfiguring, adopting Teece’s (2007) definition. Teece (2007) classified DCs into 12 major activities of clusters. Embedding the words constituting the four activities such as analyzing internal R&D, tapping external R&D, tapping supplier and complementor innovation, and identifying customer needs, which are components of Teece’s (2007) sensing of DCs, converges to two clusters of analysis and identifying. In a similar research line, Rudolph (2017) developed a codebook of DCs based on corporate performance analysis. Rudolph (2017) presented the measurement of the sensing capability by simplifying them into market-oriented and technology-oriented characteristics because it is very ambiguous to distinguish among four subcategories. Therefore, in this study, we follow Rudolph’s (2017) classification in defining DCs according to 10 detailed capabilities. Sensing can be explained by the ability to analyze and identify, and seizing represents activities that (re)design business models, select decision-making protocols, build loyalty and commitment, and define enterprise boundaries. Finally, reconfiguring is presented with a focus on appropriate knowledge management, governance, cospecialization and decentralization capabilities.

The measurement of uncertainty is as follows by referring to the theoretical model that integrates the moderating effects of environmental dynamism on sustainability and DCs. Existing researchers calculated uncertainty for changes in profitability and volatility of a certain indicator, and presented it as value representing the frequency of fluctuations in specific areas, such as product demand. Therefore, this study employs the measurement of environmental uncertainty that presents the results calculated through standard deviation in the variation rate of profit margin.

As a proxy of a dependent variable, this study employs the two measurements of financial performance to evaluate the effect of DCs on the spread of corporate performance. In particular, ROA (Return on Assets), a short-term financial performance, which is the core of profitability indicators measures economic performance as a ratio of net profit to total assets. Another proxy of financial performance is Tobin’s Q, a long-term market value, which is used as a representative market performance indicator. In the case of Tobin’s Q, various calculation methods are used to measure investors’ perception.

Tobin’s Q is a measure of a firm’s market performance and is defined as the ratio of market value divided by the firm’s asset replacement cost (Tobin, 1969). However, since it is difficult to objectively measure the market value of asset replacement cost and liabilities, Equation (1) was used to measure the value by dividing the average market capitalization by the total assets as shown below (Li and Wang, 2019).


(1) $$TBQ=\frac{marketcapitalization}{totalassets}$$

It is explained that R&D investment, one of the main activities in DCs, contributes to improving the corporate performance. This study aims to evaluate the performance improvement effect of R&D intensity by examining R&D investment in proportion to a company’s assets, rather than evaluating a company’s R&D capability with the traditionally used simple R&D investment amount (Reynard, 1979; García-Manjón and Romero-Merino, 2012). Using R&D intensity, it is possible to examine in detail whether a company is spending R&D expenses in line with its size. Therefore, as the R&D capability for this research model, R&D intensity calculated as R&D expenditure compared to operating profit was adopted.

The main factors that determine corporate performance include PER (Price to Earnings Ratio), current ratio, debt ratio, firm size, and firm age. These financial factors are generally input as control variables in a research model that uses corporate performance as a dependent variable, and the influence of predictor variables is considered. The PER, which is considered as a control variable, is an index that judges the enterprise value in the market from a short-term perspective. The evaluation of productivity, a financial performance indicator, through sales per employee was also considered in this study. The current ratio was calculated as current assets compared to current liabilities to measure a company’s ability to pay its debts. The debt ratio is calculated as the debt-to-asset ratio to measure a company’s risk.

Firm size is measured by the number of employees or total assets are used as proxies. In this study, total assets were replaced with natural logarithms to control the firm size. When the number of employees was employed as a proxy for firm size, the multicollinearity problem occurred, and thus total assets substituted with natural logarithms were adopted as the final proxy of firm size. Given that high-tech industries specialize in intangible assets, when total assets are adopted as a proxy for firm size, measurement errors may occur. In contrast to this, there will be less error in asset measurement, so consistency can be maintained since the general manufacturing and service companies targeted in this study have a greater proportion of tangible assets to intangible assets. The firm age was calculated by taking the natural logarithm of the number of years of business activity since the establishment of the company. Ultimately, in order to control the impact of industry on corporate financial performance, it was analyzed by treating it as a dummy variable.

### Research Model

In this section, we will explain the model description for the empirical analysis and the characteristics of the key variable data used in the model. Since the collected panel data are cross-sectional containing time series data and characteristics of each company, the predictive power of a panel model analysis is higher than that of multiple regression analysis (Baltagi et al., 2005; Hsiao, 2007). In other words, the panel data has a high model fit because it can reflect the dynamic relationship as well as the characteristics of multiple objects because the object is repeatedly observed over time (Baltagi et al., 2005; Hsiao, 2007). In an empirical analysis, the researcher must select a model suitable for his or her research framework between fixed-effect and random-effect models.

In the process of securing ESG continuous data, a lot of data preprocessing is required. In general, it is rare for all data to be uniformly distributed. In most cases, the data is imbalanced due to missing items or missing values at a specific point in time. In this study, global 500 companies were investigated, and 10 years of balanced panel data of 93 companies was finally obtained. In order to analyze these fixed panel data, a fixed-effect panel model should be adopted (Baltagi et al., 2005; Baum, 2006). Therefore, this study established a final research model that examines the relationship between a company’s DCs and its financial performance through a fixed-effect panel model.

In this study, the financial performance of a company was divided into two types. One is a research model with corporate performance (ROA) as a dependent variable, and the other is a regression model with long-term corporate value (Tobin’s Q) as a dependent variable. DCs, the main variable in this research model, are independent variables that can examine the strategic characteristics of a company. Control variables that affect the dependent variable include uncertainty, R&D intensity, PER, potential growth, liquidity, debt ratio, firm size, and firm age. Also, the interaction term between DCs and uncertainty variables was examined. The equation for performing the hypothesis test in this study is as follows.


(2) $$\begin{array}{c} {\text{y}}_{i,t}=\alpha +\beta_{1}ANL_{i,t}+\beta_{2}IDN_{i,t}+\beta_{3}EBR_{i,t}+\beta_{4}BMD_{i,t}\\ +\beta_{5}DMP_{i,t}+\beta_{6}LYC_{i,t}+\beta_{7}COS_{i,t}+\beta_{8}GOV_{i,t}\\ +\beta_{9}KNW_{i,t}+\beta_{10}DEC_{i,t}+\beta_{11}RND_{i,t}+\beta_{12}PER_{i,t}\\ +\beta_{13}RPE_{i,t}+\beta_{14}CR_{i,t}+\beta_{15}DB_{i,t}+\beta_{16}FS_{i,t}\\ +\beta_{17}AG_{i,t}+\varepsilon_{i,t} \end{array}$$

(3) $$\begin{array}{c} {\text{y}}_{i,t}=\alpha +\beta_{K}DCs_{i,tK}+\beta_{11}UNC_{i,t}+\beta_{12}RND_{i,t}+\beta_{13}PER_{i,t}\\ +\beta_{14}RPE_{i,t}+\beta_{15}CR_{i,t}+\beta_{16}DB_{i,t}+\beta_{17}FS_{i,t}\\ +\beta_{18}AG_{i,t}+\varepsilon_{i,t} \end{array}$$

(4) $$\begin{array}{c} {\text{y}}_{i,t}=\alpha +\beta_{K}DCs_{i,tK}+\beta_{11}UNC_{i,t}+\beta_{K}DCs_{i,tK}^{*}UNC_{i,tK}\\ +\beta_{22}RND_{i,t}+\beta_{23}PER_{i,t}+\beta_{24}RPE_{i,t}+\beta_{25}CR_{i,t}\\ +\beta_{26}DB_{i,t}+\beta_{27}FS_{i,t}+\beta_{28}AG_{i,t}+\varepsilon_{i,t} \end{array}$$

*where, DC refers to dynamic capabilities, RND refers to R&D intensity, PER refers to price to earnings ratio, RPE refers to revenue per employee, CR refers to current ratio, DB refers to debt ratio, FS refers to firm size, AG refers to firm age, UNC refers to uncertainty, ε is an error term, and i indicates an entity, t indicates time, and k indicates 1,…,10.*

The first step equation (Equation 2) reveals the correlation between DCs and corporate performance. The second step equation (Equation 3) adds uncertainty to the first step equation. In the final model (Equation 4), a fixed-effect panel analysis is performed by inputting firm specific attributes including the interaction terms of DCs and uncertainty and financial items to analyze the moderating effect.

## Empirical Results

### Descriptive Analysis

Table 3 shows the descriptive statistics of DCs and major variables used in the research model. In descriptive statistics, the mean, standard deviation, maximum, and minimum values are presented as the basis for data normality. As a method to determine whether each variable follows a multivariate normal distribution, the standards of skewness and kurtosis are reviewed from Curran et al. (1996). In general, when skewness is more than ± 2 and kurtosis is not more than ± 7, it is judged that it does not affect the estimation. In this study, kurtosis and skewness did not exceed the reference values, so there was no problem with normality. Also, to verify the reliability of the scale shown by each indicator, we looked at the Cronbach alpha value. The Cronbach alpha value of all scales was 0.6 or higher, indicating a value satisfactory for reliability (Cronbach, 1951; Sijtsma, 2009; Kiliç, 2016).

**TABLE 3:** Descriptive statistics and measures of skewness, kurtosis test for variables.

Variables	Mean	P50	Min	Max	S.D.	Kurtosis	Skewness
ROA	12.082	10.385	0.190	47.910	7.882	6.006	1.400
TBQ	0.905	0.612	0.010	5.100	0.929	6.891	1.807
ANL	0.145	0.097	0.004	0.855	0.141	6.082	1.635
IDN	0.148	0.107	0.004	0.828	0.134	5.241	1.410
EBR	0.170	0.143	0.004	0.880	0.143	5.241	1.410
BMD	0.169	0.141	0.004	0.888	0.140	6.043	1.505
DMP	0.148	0.108	0.003	0.835	0.137	6.853	1.664
LYC	0.165	0.135	0.004	0.931	0.133	6.063	1.349
COS	0.167	0.140	0.003	0.844	0.134	4.889	1.169
GOV	0.182	0.159	0.003	0.764	0.135	4.161	1.022
KNW	0.155	0.135	0.003	0.817	0.121	5.293	1.137
DEC	0.174	0.149	0.004	0.845	0.136	4.758	1.172
UNC	0.14	0.06	0.00	2.62	0.25	6.604	1.400
RND	5.08	2.75	0.01	44.13	6.51	6.242	1.913
PER	26.07	15.13	0.30	770.52	63.14	6.650	1.884
RPE	1879	618	86	27993	3552	6.572	1.816
CR	1.322	1.210	0.170	5.530	0.647	4.833	1.748
DB	68.02	69.39	27.71	114.23	15.51	2.645	0.056
FS	17.30	17.83	9.14	22.32	2.57	3.846	-1.077
AG	44.22	30.00	2.00	155.00	35.60	3.582	1.154

*ROA, return on assets; TBQ, Tobin’s Q; ANL, analyzing; IDN, identifying; EBR, enterprise boundaries; BMD, business model design; DMP, decision-making protocols; LYC, loyalty and commitment; COS, cospecialization; GOV, governance; KNW, knowledge management; DEC, decentralization; UNC, uncertainty; RND, R&D intensity; PER, price earnings ratio; RPE, revenue per employee; CR, current ratio; DB, debt ratio; FS, firm size; AG, firm age.*

### Correlation Analysis

In this study, the Pearson correlation analysis of variables was performed, and the results are shown in Table 4. Variables with correlation coefficients greater than 0.5 among various variables in DCs are: in the analysis of the sensing group, the correlation between analysis and identifying was 0.562, the correlation between enterprise boundaries and loyalty and commitment in the seizing group was 0.598, and the correlation between cospecialization and decentralization in the reconfiguring group was 0.591. All cases showed a rather high correlation coefficient, but it was confirmed to be smaller than the general cut-off value of 0.7.

**TABLE 4:** Bivariate correlations matrix.

	VIF	1	2	3	4	5	6	7	8	9	10	11	12	13	14	15	16	17	18	19	20
ROA	1.103	1																			
TBQ	1.121	0.187	1																		
ANL	1.739	–0.001	0.018	1																	
IDN	1.874	0.070	–0.019	0.562	1																
EBR	2.063	0.019	0.039	0.237	0.464	1															
BMD	1.651	0.007	–0.031	0.115	0.165	0.419	1														
DMP	1.661	0.042	0.063	0.166	0.180	0.582	0.448	1													
LYC	1.827	0.000	–0.022	0.220	0.234	0.598	0.412	0.438	1												
COS	2.479	0.056	–0.067	0.194	0.145	0.143	0.294	0.230	0.270	1											
GOV	2.677	0.044	0.023	0.249	0.295	0.227	0.459	0.139	0.180	0.558	1										
KNW	2.406	–0.021	–0.039	0.152	0.292	0.242	0.242	0.183	0.106	0.511	0.582	1									
DEC	2.692	0.066	–0.023	0.197	0.205	0.255	0.298	0.270	0.267	0.591	0.526	0.543	1								
UNC	1.093	–0.121	–0.076	–0.046	–0.007	–0.052	–0.019	–0.054	–0.028	–0.010	–0.054	–0.062	–0.052	1							
RND	1.124	0.045	0.127	–0.088	–0.022	0.088	0.001	0.094	–0.032	–0.060	–0.122	–0.052	–0.077	–0.048	1						
PER	1.110	–0.036	0.172	–0.044	–0.057	–0.042	–0.102	–0.036	–0.040	–0.045	0.013	–0.051	–0.069	0.008	0.048	1					
RPE	1.161	–0.097	–0.159	–0.051	0.006	0.000	0.040	0.058	0.008	–0.044	–0.114	–0.051	–0.070	0.153	0.010	–0.021	1				
CR	1.138	0.131	0.074	0.020	0.044	0.031	–0.037	0.017	0.036	0.046	0.035	0.017	0.001	–0.133	–0.090	–0.007	0.000	1			
DB	1.226	–0.067	0.147	0.005	0.088	0.019	–0.005	–0.051	–0.043	0.047	0.044	0.056	0.046	0.042	0.095	0.137	–0.141	–0.250	1		
FS	1.213	–0.023	–0.229	–0.053	–0.006	0.002	0.048	0.075	–0.001	–0.032	–0.101	–0.013	–0.015	–0.105	–0.035	–0.153	0.117	0.018	–0.202	1	
AG	1.097	–0.072	0.013	–0.006	0.013	–0.036	0.011	–0.003	–0.022	0.031	0.050	0.061	0.059	–0.004	–0.154	0.042	–0.115	0.046	–0.118	–0.113	1

*ROA, return on assets; TBQ, Tobin’s Q; ANL, analyzing; IDN, identifying; EBR, enterprise boundaries; BMD, business model design; DMP, decision-making protocols; LYC, loyalty and commitment; COS, cospecialization; GOV, governance; KNW, knowledge management; DEC, decentralization; UNC, uncertainty; RND, R&D intensity; PER, price earnings ratio; RPE, revenue per employee; CR, current ratio; DB, debt ratio; FS, firm size; AG, firm age.*

In addition, the Variance Inflation Factors (VIF) value was estimated for an additional multicollinearity test before the research model estimation was performed, and the average VIF value of all variables was less than 3.3. Therefore, it was concluded that the research model of this study is not affected by the multicollinearity problem (Diamantopoulos and Siguaw, 2006; Petter et al., 2007; Cenfetelli and Bassellier, 2009). In this study, the Hausman Test, a representative test that judges which model is more reliable among the fixed-effect model and the random-effect model (Hausman, 1978). As a result of Hausman Test conducted on the final models (Model 16 and Model 32) in Tables 5 and 6, respectively, the *P*-value was all 0.00001. That is, since *P* < 0.05, it can be judged that the fixed effect model is a reliable model.

**TABLE 5:** Fixed effect panel model estimation of the impact of DCV and environmental uncertainty on ROA.

	Model 1	Model 2	Model 3	Model 4	Model 5	Model 6	Model 7	Model 8	Model 9	Model 10	Model 11	Model 12	Model 13	Model 14	Model 15	Model 16
ANL	–0.040	–0.091	0.550	0.058	–0.023	–0.049	–0.095	–0.094	–0.075	–0.176	–0.140	–0.290	–1.008	–0.065	–0.030	–1.082
	(1.540)	(1.542)	(1.808)	(1.543)	(1.547)	(1.541)	(1.546)	(1.553)	(1.552)	(1.544)	(1.555)	(1.552)	(2.036)	(1.558)	(1.559)	(2.110)
IDN	2.528*	2.447*	2.389	3.461**	2.402	2.340	2.448*	2.448*	2.445*	2.403	2.468*	2.471*	4.153**	2.342	2.193	4.183**
	(1.460)	(1.464)	(1.468)	(1.594)	(1.467)	(1.466)	(1.466)	(1.466)	(1.466)	(1.465)	(1.468)	(1.464)	(1.812)	(1.471)	(1.475)	(1.867)
EBR	−2.820*	−2.848*	−2.802*	−2.792*	–2.318	−2.608*	−2.844*	−2.849*	−2.852*	−2.826*	−2.852*	−2.884*	−2.843*	–2.946	−2.935*	−3.964**
	(1.538)	(1.539)	(1.541)	(1.537)	(1.769)	(1.549)	(1.544)	(1.541)	(1.541)	(1.539)	(1.540)	(1.539)	(1.539)	(1.889)	(1.541)	(1.973)
BMD	–0.032	–0.111	–0.082	–0.107	–0.108	0.918	–0.114	–0.112	–0.103	–0.126	–0.136	–0.169	–0.156	1.199	0.077	1.373
	(1.302)	(1.306)	(1.307)	(1.304)	(1.307)	(1.528)	(1.309)	(1.310)	(1.309)	(1.306)	(1.310)	(1.307)	(1.306)	(1.724)	(1.312)	(1.775)
DMP	−3.082*	−3.035*	−3.000*	−2.872*	−3.010*	−3.112*	–2.998	−3.033*	−3.044*	−2.952*	−2.994*	−2.883*	−2.839*	−3.270*	−3.006*	−3.730*
	(1.667)	(1.668)	(1.670)	(1.669)	(1.670)	(1.668)	(1.861)	(1.672)	(1.672)	(1.670)	(1.677)	(1.674)	(1.670)	(1.923)	(1.677)	(1.945)
LYC	2.363	2.426	2.385	2.277	2.362	2.286	2.429	2.438	2.422	2.435	2.444	2.563*	2.264	2.238	2.563*	2.234
	(1.539)	(1.541)	(1.544)	(1.542)	(1.546)	(1.544)	(1.544)	(1.696)	(1.544)	(1.541)	(1.544)	(1.546)	(1.543)	(1.744)	(1.552)	(1.765)
COS	–2.890	–2.870	–2.959	–2.962	–2.881	–2.981	–2.874	–2.871	–2.932	–2.985	–2.876	–3.050	–2.855	–2.983	−4.644**	−4.233*
	(1.859)	(1.860)	(1.865)	(1.858)	(1.861)	(1.861)	(1.864)	(1.864)	(1.955)	(1.862)	(1.862)	(1.867)	(1.863)	(1.869)	(2.169)	(2.200)
GOV	1.975	2.018	2.070	1.875	2.057	2.162	2.020	2.021	1.998	3.242	2.093	2.344	1.698	2.155	4.172*	3.887
	(1.952)	(1.954)	(1.956)	(1.953)	(1.956)	(1.955)	(1.956)	(1.962)	(1.965)	(2.251)	(1.976)	(1.976)	(1.966)	(1.969)	(2.514)	(2.590)
KNW	–2.146	–2.193	–2.187	–1.981	–2.171	–2.212	–2.191	–2.192	–2.216	–2.121	–1.986	–2.145	–1.870	–2.247	−4.201*	−4.238*
	(2.048)	(2.049)	(2.050)	(2.050)	(2.051)	(2.048)	(2.052)	(2.053)	(2.063)	(2.050)	(2.199)	(2.049)	(2.056)	(2.057)	(2.544)	(2.558)
DEC	2.744	2.790	2.719	2.703	2.766	2.782	2.793	2.790	2.794	2.747	2.778	3.606*	2.781	2.791	5.001*	5.068*
	(2.017)	(2.019)	(2.022)	(2.016)	(2.020)	(2.017)	(2.021)	(2.021)	(2.021)	(2.019)	(2.021)	(2.151)	(2.019)	(2.027)	(2.556)	(2.632)
UNC		–0.559	–0.181	0.325	–0.027	0.660	–0.525	–0.546	–0.712	0.876	–0.244	0.836	0.157	0.415	0.168	1.128
		(0.695)	(0.891)	(0.887)	(1.118)	(1.172)	(1.029)	(1.111)	(1.638)	(1.485)	(1.391)	(1.450)	(0.912)	(1.529)	(1.686)	(2.010)
UNC × ANL			–5.566										10.014			11.129
			(8.180)										(12.466)			(13.127)
UNC × IDN				–11.463									−18.108*			−21.969*
				(7.168)									(10.947)			(11.832)
UNC × EBR					–3.715									2.737		8.960
					(6.105)									(9.254)		(10.476)
UNC × BMD						–8.460								–10.656		–10.577
						(6.544)								(9.097)		(9.820)
UNC × DMP							–0.322							0.979		6.906
							(7.109)							(8.881)		(9.471)
UNC × LYC								–0.078						0.368		0.432
								(4.883)						(5.962)		(6.505)
UNC × COS									0.712						13.114	8.074
									(6.915)						(11.175)	(12.125)
UNC × GOV										–6.962					–13.353	–13.548
										(6.368)					(11.239)	(12.301)
UNC × KNW											–1.792				16.108	20.036
											(6.839)				(14.370)	(14.638)
UNC × DEC												–7.356			–19.100	–19.972
												(6.710)			(13.441)	(14.731)
RND	−0.671***	−0.674***	−0.673***	−0.675***	−0.674***	−0.674***	−0.674***	−0.674***	−0.675***	−0.667***	−0.674***	−0.671***	−0.679***	−0.675***	−0.667***	−0.672***
	(0.083)	(0.083)	(0.083)	(0.083)	(0.083)	(0.083)	(0.083)	(0.083)	(0.083)	(0.083)	(0.083)	(0.083)	(0.083)	(0.083)	(0.084)	(0.084)
PER	−0.009***	−0.009***	−0.009***	−0.009***	−0.009***	−0.009***	−0.009***	−0.009***	−0.009***	−0.009***	−0.009***	−0.009***	−0.009***	−0.009***	−0.009***	−0.009***
	(0.002)	(0.002)	(0.002)	(0.002)	(0.002)	(0.002)	(0.002)	(0.002)	(0.002)	(0.002)	(0.002)	(0.002)	(0.002)	(0.002)	(0.002)	(0.002)
RPE	0.000	0.000	0.000	0.000	0.000	0.000	0.000	0.000	0.000	0.000	0.000	0.000	0.000	0.000	0.000	0.000
	(0.000)	(0.000)	(0.000)	(0.000)	(0.000)	(0.000)	(0.000)	(0.000)	(0.000)	(0.000)	(0.000)	(0.000)	(0.000)	(0.000)	(0.000)	(0.000)
CR	–0.275	–0.263	–0.258	–0.253	–0.254	–0.247	–0.262	–0.263	–0.262	–0.261	–0.262	–0.256	–0.256	–0.250	–0.240	–0.241
	(0.267)	(0.267)	(0.268)	(0.267)	(0.268)	(0.267)	(0.268)	(0.268)	(0.268)	(0.267)	(0.268)	(0.267)	(0.267)	(0.268)	(0.267)	(0.268)
DB	–0.040	–0.038	–0.037	–0.039	–0.037	–0.037	–0.038	–0.038	–0.037	–0.039	–0.038	–0.039	–0.040	–0.037	–0.037	–0.041
	(0.029)	(0.029)	(0.029)	(0.029)	(0.029)	(0.029)	(0.029)	(0.029)	(0.029)	(0.029)	(0.029)	(0.029)	(0.029)	(0.029)	(0.029)	(0.029)
FS	0.660	0.677	0.655	0.677	0.698	0.655	0.676	0.677	0.671	0.785	0.695	0.740	0.717	0.641	0.765	0.777
	(0.488)	(0.488)	(0.490)	(0.488)	(0.490)	(0.488)	(0.490)	(0.491)	(0.493)	(0.498)	(0.494)	(0.492)	(0.490)	(0.496)	(0.499)	(0.508)
AG	−0.181***	−0.182***	−0.180***	−0.178***	−0.183***	−0.183***	−0.182***	−0.182***	−0.181***	−0.195***	−0.184***	−0.191***	−0.180***	−0.182***	−0.192***	−0.191***
	(0.065)	(0.065)	(0.065)	(0.065)	(0.065)	(0.065)	(0.065)	(0.065)	(0.065)	(0.066)	(0.065)	(0.065)	(0.065)	(0.065)	(0.066)	(0.066)
Industry dummies	YES	YES	YES	YES	YES	YES	YES	YES	YES	YES	YES	YES	YES	YES	YES	YES
_cons	11.818	11.498	11.680	11.290	11.107	11.716	11.507	11.509	11.571	10.129	11.264	10.693	10.842	11.980	10.238	10.283
	(7.560)	(7.574)	(7.582)	(7.563)	(7.606)	(7.570)	(7.584)	(7.611)	(7.615)	(7.675)	(7.633)	(7.608)	(7.586)	(7.676)	(7.679)	(7.789)
N	580	580	580	580	580	580	580	580	580	580	580	580	580	580	580	580
R^2^	0.244	0.245	0.246	0.249	0.246	0.248	0.245	0.245	0.245	0.247	0.245	0.247	0.250	0.248	0.253	0.260
F	13.400	13.200	13.150	13.190	13.170	13.100	13.120	13.130	13.130	13.060	13.130	13.020	13.190	12.980	12.950	12.830
Hausman (Prob > χ^2^)	0.0000	0.0000	0.0000	0.0000	0.0000	0.0000	0.0000	0.0000	0.0000	0.0000	0.0000	0.0000	0.0000	0.0000	0.0000	0.0000

***, **, and * refer to significance at 1%, 5%, and 10%, respectively. ROA, return on assets; TBQ, Tobin’s Q; ANL, analyzing; IDN, identifying; EBR, enterprise boundaries; BMD, business model design; DMP, decision-making protocols; LYC, loyalty and commitment; COS, cospecialization; GOV, governance; KNW, Knowledge management; DEC, decentralization; UNC, uncertainty; RND, R&D intensity; PER, price earnings ratio; RPE, revenue per employee; CR, current ratio; DB, debt ratio; FS, firm size; AG, firm age.

**TABLE 6:** Fixed effect panel model estimation of the impact of DCV and environmental uncertainty on Tobin’s Q.

	Model 17	Model 18	Model 19	Model 20	Model 21	Model 22	Model 23	Model 24	Model 25	Model 26	Model 27	Model 28	Model 29	Model 30	Model 31	Model 32
ANL	–0.127	–0.130	0.098	–0.105	–0.123	–0.129	–0.132	–0.122	–0.122	–0.131	–0.118	–0.151	–0.016	–0.118	–0.127	0.056
	(0.187)	(0.188)	(0.219)	(0.187)	(0.188)	(0.188)	(0.188)	(0.189)	(0.189)	(0.188)	(0.189)	(0.189)	(0.247)	(0.190)	(0.189)	(0.256)
IDN	0.455**	0.449**	0.429**	0.616***	0.445**	0.448**	0.450**	0.448**	0.448**	0.449**	0.444**	0.452**	0.558**	0.447**	0.417**	0.518**
	(0.178)	(0.178)	(0.178)	(0.193)	(0.179)	(0.179)	(0.178)	(0.178)	(0.178)	(0.178)	(0.179)	(0.178)	(0.220)	(0.179)	(0.179)	(0.226)
EBR	−0.474**	−0.476**	−0.460**	−0.467**	−0.425**	−0.474**	−0.474**	−0.474**	−0.478**	−0.476**	−0.475**	−0.480**	−0.463**	−0.419*	−0.494***	−0.494**
	(0.187)	(0.187)	(0.187)	(0.187)	(0.215)	(0.189)	(0.188)	(0.188)	(0.187)	(0.187)	(0.187)	(0.187)	(0.187)	(0.230)	(0.187)	(0.239)
BMD	0.046	0.041	0.051	0.042	0.041	0.052	0.040	0.045	0.045	0.041	0.047	0.035	0.046	0.015	0.068	0.057
	(0.158)	(0.159)	(0.158)	(0.158)	(0.159)	(0.186)	(0.159)	(0.159)	(0.159)	(0.159)	(0.159)	(0.159)	(0.159)	(0.210)	(0.159)	(0.215)
DMP	0.025	0.028	0.040	0.055	0.030	0.027	0.047	0.024	0.024	0.029	0.018	0.044	0.052	0.054	0.026	–0.074
	(0.203)	(0.203)	(0.202)	(0.203)	(0.203)	(0.203)	(0.226)	(0.203)	(0.203)	(0.203)	(0.204)	(0.204)	(0.203)	(0.234)	(0.203)	(0.235)
LYC	0.118	0.122	0.108	0.098	0.116	0.121	0.124	0.091	0.121	0.123	0.118	0.137	0.099	0.088	0.160	0.077
	(0.187)	(0.188)	(0.187)	(0.187)	(0.188)	(0.188)	(0.188)	(0.206)	(0.188)	(0.188)	(0.188)	(0.188)	(0.187)	(0.212)	(0.188)	(0.214)
COS	−0.504**	−0.503**	−0.534**	−0.518**	−0.504**	−0.504**	−0.505**	−0.499**	−0.532**	−0.505**	−0.502**	−0.522**	−0.527**	−0.500**	−0.692***	−0.702***
	(0.226)	(0.226)	(0.226)	(0.226)	(0.226)	(0.227)	(0.227)	(0.227)	(0.238)	(0.227)	(0.226)	(0.227)	(0.226)	(0.228)	(0.263)	(0.266)
GOV	–0.044	–0.042	–0.023	–0.065	–0.038	–0.040	–0.041	–0.049	–0.051	–0.020	–0.060	–0.006	–0.050	–0.048	0.022	–0.023
	(0.237)	(0.238)	(0.237)	(0.237)	(0.238)	(0.238)	(0.238)	(0.239)	(0.239)	(0.274)	(0.240)	(0.240)	(0.239)	(0.240)	(0.305)	(0.314)
KNW	−0.606**	−0.609**	−0.607**	−0.574**	−0.607**	−0.609**	−0.608**	−0.613**	−0.620**	−0.608**	−0.660**	−0.604**	−0.584**	−0.608**	−1.017***	−1.021***
	(0.249)	(0.249)	(0.249)	(0.249)	(0.250)	(0.250)	(0.250)	(0.250)	(0.251)	(0.250)	(0.268)	(0.249)	(0.250)	(0.251)	(0.309)	(0.310)
DEC	0.850***	0.853***	0.828***	0.839***	0.851***	0.853***	0.854***	0.854***	0.855***	0.852***	0.856***	0.941***	0.832***	0.853***	1.345***	1.394***
	(0.245)	(0.246)	(0.245)	(0.245)	(0.246)	(0.246)	(0.246)	(0.246)	(0.246)	(0.246)	(0.246)	(0.262)	(0.245)	(0.247)	(0.310)	(0.319)
UNC		–0.036	0.098	0.109	0.015	–0.024	–0.019	–0.075	–0.108	–0.012	–0.114	0.114	0.123	–0.029	–0.022	0.011
		(0.085)	(0.108)	(0.108)	(0.136)	(0.143)	(0.125)	(0.135)	(0.199)	(0.181)	(0.169)	(0.176)	(0.111)	(0.186)	(0.205)	(0.243)
UNC × ANL			−1.977**										–0.835			–1.328
			(0.992)										(1.514)			(1.590)
UNC × IDN				−1.882**									–1.328			–1.296
				(0.870)									(1.330)			(1.433)
UNC × EBR					–0.361									–0.406		–0.038
					(0.743)									(1.128)		(1.269)
UNC × BMD						–0.087								0.233		0.273
						(0.798)								(1.109)		(1.189)
UNC × DMP							–0.167							–0.219		1.131
							(0.865)							(1.082)		(1.147)
UNC × LYC								0.217						0.228		0.444
								(0.594)						(0.726)		(0.788)
UNC × COS									0.334						1.063	0.960
									(0.841)						(1.356)	(1.469)
UNC × GOV										–0.120					–0.310	–0.049
										(0.776)					(1.364)	(1.490)
UNC × KNW											0.438				3.500**	3.671**
											(0.832)				(1.744)	(1.773)
UNC × DEC												–0.794			−4.191**	−4.917***
												(0.817)			(1.631)	(1.784)
RND	−0.036***	−0.036***	−0.035***	−0.036***	−0.036***	−0.036***	−0.036***	−0.036***	−0.036***	−0.036***	−0.036***	−0.035***	−0.036***	−0.036***	−0.036***	−0.036***
	(0.010)	(0.010)	(0.010)	(0.010)	(0.010)	(0.010)	(0.010)	(0.010)	(0.010)	(0.010)	(0.010)	(0.010)	(0.010)	(0.010)	(0.010)	(0.010)
PER	−0.001***	−0.001***	−0.001***	−0.001***	−0.001***	−0.001***	−0.001***	−0.001***	−0.001***	−0.001***	−0.001***	−0.001***	−0.001***	−0.001***	−0.001***	−0.001***
	(0.000)	(0.000)	(0.000)	(0.000)	(0.000)	(0.000)	(0.000)	(0.000)	(0.000)	(0.000)	(0.000)	(0.000)	(0.000)	(0.000)	(0.000)	(0.000)
RPE	–0.000	–0.000	–0.000	–0.000	–0.000	–0.000	–0.000	–0.000	–0.000	–0.000	–0.000	–0.000	–0.000	–0.000	–0.000	–0.000
	(0.000)	(0.000)	(0.000)	(0.000)	(0.000)	(0.000)	(0.000)	(0.000)	(0.000)	(0.000)	(0.000)	(0.000)	(0.000)	(0.000)	(0.000)	(0.000)
CR	–0.043	–0.042	–0.040	–0.040	–0.041	–0.042	–0.042	–0.042	–0.042	–0.042	–0.042	–0.041	–0.040	–0.041	–0.038	–0.037
	(0.032)	(0.033)	(0.032)	(0.032)	(0.033)	(0.033)	(0.033)	(0.033)	(0.033)	(0.033)	(0.033)	(0.033)	(0.032)	(0.033)	(0.032)	(0.032)
DB	−0.009**	−0.009**	−0.008**	−0.009**	−0.009**	−0.009**	−0.009**	−0.009**	−0.008**	−0.009**	−0.009**	−0.009**	−0.009**	−0.009**	−0.008**	−0.008**
	(0.003)	(0.003)	(0.003)	(0.003)	(0.003)	(0.003)	(0.003)	(0.003)	(0.004)	(0.004)	(0.003)	(0.003)	(0.003)	(0.004)	(0.004)	(0.004)
FS	−0.230***	−0.229***	−0.237***	−0.229***	−0.227***	−0.229***	−0.230***	−0.227***	−0.232***	−0.227***	−0.234***	−0.222***	−0.233***	−0.225***	−0.234***	−0.228***
	(0.059)	(0.059)	(0.059)	(0.059)	(0.060)	(0.060)	(0.060)	(0.060)	(0.060)	(0.061)	(0.060)	(0.060)	(0.060)	(0.060)	(0.061)	(0.062)
AG	0.044***	0.044***	0.045***	0.045***	0.044***	0.044***	0.044***	0.044***	0.044***	0.044***	0.045***	0.043***	0.045***	0.044***	0.044***	0.044***
	(0.008)	(0.008)	(0.008)	(0.008)	(0.008)	(0.008)	(0.008)	(0.008)	(0.008)	(0.008)	(0.008)	(0.008)	(0.008)	(0.008)	(0.008)	(0.008)
Industry dummies	YES	YES	YES	YES	YES	YES	YES	YES	YES	YES	YES	YES	YES	YES	YES	YES
_cons	3.746***	3.725***	3.790***	3.691***	3.687***	3.727***	3.730***	3.695***	3.760***	3.702***	3.782***	3.638***	3.728***	3.652***	3.772***	3.670***
	(0.919)	(0.921)	(0.919)	(0.918)	(0.925)	(0.922)	(0.922)	(0.926)	(0.926)	(0.935)	(0.928)	(0.926)	(0.921)	(0.935)	(0.931)	(0.943)
*N*	580	580	580	580	580	580	580	580	580	580	580	580	580	580	580	580
*R* ^2^	0.141	0.141	0.148	0.149	0.142	0.141	0.141	0.141	0.141	0.141	0.142	0.143	0.150	0.142	0.155	0.166
*F*	28.140	27.650	27.450	27.800	27.590	27.500	27.540	27.490	27.610	27.480	27.520	27.590	27.420	27.250	27.660	27.380
Hausman (Prob > χ^2^)	0.0000	0.0000	0.0000	0.0000	0.0000	0.0000	0.0000	0.0000	0.0000	0.0000	0.0000	0.0000	0.0000	0.0000	0.0000	0.0000

***, **, and * refer to significance at 1%, 5%, and 10%, respectively. ROA, return on assets; TBQ, Tobin’s Q; ANL, analyzing; IDN, identifying; EBR, enterprise boundaries; BMD, business model design; DMP, decision-making protocols; LYC, loyalty and commitment; COS, cospecialization; GOV, governance; KNW, knowledge management; DEC, decentralization; UNC, uncertainty; RND, R&D intensity; PER, price earnings ratio; RPE, revenue per employee; CR, current ratio; DB, debt ratio; FS, firm size; AG, firm age.

### Hypotheses Testing

Table 5 shows the results of regression analysis using ROA as the dependent variable, and Table 6 shows the estimation results of the research model using Tobin’s Q as the dependent variable. In the empirical analysis of this study, some results were consistent with the theories and hypotheses, but in a specific model, the opposite results were also found. We should pay attention to two areas in the results of this empirical analysis: (1) the relationship between DCs and financial performance shown in the ESG reporting and (2) the moderating effect of environmental uncertainty on the DCs-performance relationship.

Among the sub-activities of DCs, the identifying capability (hypothesis 2) and the decentralization capability (hypothesis 10) showed a significant positive relationship in the regression model using corporate performance (ROA) and corporate value (Tobin’s Q) as dependent variables. Also, in part, it was found that the loyalty and commitment capability (hypothesis 6) and the governance capability (hypothesis 8) had no effect on ROA.

Looking at the statistical results, Hypothesis 2 was supported as the identifying capability had a significant positive effect on both ROA (beta = 4.183 in Model 16) and Tobin’s Q (beta = 0.518 in Model 32). Looking at research model 32 using Tobin’s Q as a dependent variable, the decentralization capability was found to be positively linked with corporate performance, supporting hypothesis 10 (beta = 1.394). Also, in Model 15 using ROA as a dependent variable, it was found that the loyalty and commitment capability (beta = 2.563) and the governance capability had a non-significant positive relationship with financial performance, so Hypothesis 6 and Hypothesis 8 were not supported.

The results of the analyzing of the direct effects of DCs on corporate performance, meanwhile, are as follows: the capabilities of enterprise boundaries (hypothesis 3), decision-making protocols (hypothesis 5), cospecialization (hypothesis 7), and knowledge management (hypothesis 9) were shown to have a significant negative relationship with corporate performance (ROA or Tobin’s Q).

Statistical results show that the selecting enterprise boundaries capability has a significant negative relationship for the two dependent variables with ROA (beta = −3.964 in Model 16) and Tobin’s Q (beta = −0.494 in Model 32). Looking at research model 32 using Tobin’s Q as a dependent variable, it was found that the cospecialization capability (beta = -0.702) and the knowledge management capability (beta = −1.021) had a significant negative correlation with corporate performance. In Model 16 using ROA as a dependent variable, the decision-making protocols capability (beta = −3.730) had a significant negative effect on financial performance.

This study presents the empirical analysis results by subdividing the dimensions of sensing, seizing, and reconfiguring, which are the three major components of Teece’s (2007) DCs. Capabilities that directly and significantly positively correlated corporate performance were the identifying and the decentralization capabilities. In addition, it was found that the loyalty and commitment and the governance capabilities have a positive effect on ROA when environmental uncertainty plays the role of a moderator. On the other hand, the results suggest that the enterprise boundaries, the decision-making protocols, the cospecialization, and the knowledge management capabilities have a negative relationship with financial performance. The analysis and the business model design capabilities, which are the main variables of the hypotheses not supported in this study, show the following characteristics: looking at the values of the correlation coefficients from Models 1 to 16 that use ROA as a dependent variable, it is possible to find that the direction is irregular. For example, in Model 1, the correlation coefficient value is negative, but in Model 3, on the contrary, it appears positive, indicating an inconsistency of results.

Regarding Hypothesis 11, the various relationships of DCs with corporate performance under uncertainty found in this study are as follows. First, the direct effect of the identifying capability showed a generally positive relationship in the research model in which corporate performance (ROA) and corporate value (Tobin’s Q) were dependent variables. However, the result was changed to a negative relationship through interaction with environmental uncertainty on the dependent variable ROA (beta = −21.969 in Model 16).

Second, the direct effect of the knowledge management capability showed a negative relationship in the research model in which the corporate value (Tobin’s Q) was a dependent variable. However, in the interaction with environmental uncertainty, the result was reversed to a positive relationship (beta = 3.671 in Model 32). Finally, the direct effect of the decentralization capability showed a positive relationship in the research model in which the corporate value (Tobin’s Q) was the dependent variable. However, the result was reversed to a negative relationship due to the interaction effect with environmental uncertainty (beta = −4.917 in Model 32).

### Robustness Test

The results of the empirical analysis should secure consistency through a robustness test. In this study, three methods were adopted for the robustness test: inputting control variables sequentially, replacing different proxies for the same variable, and verifying first-order autocorrelation of the error term. First, by introducing sequential control variables, we tried to avoid methodological distortion that may occur when simultaneously inputting control variables. As a result of the test, there was a slight difference between the statistical significance and the regression coefficient, but the positive or negative direction indicating the relationship to the dependent variable did not change. Second, alternative proxies for the same variables were adopted to examine the consistency of the analysis results, and other proxies for growth, productivity, and activity were substituted. As a result of the test, there was no significant change in the direction of the regression coefficient and the statistical significance. Finally, the Bhargava test (Bhargava et al., 1982) and the Baltagi–Wu test (Baltagi and Wu, 1999) were performed to determine whether there was a first-order autocorrelation of the error term. Each test statistic showed a value close to the threshold of 2, for the 5% significance level, so the null hypothesis, i.e., there is no autocorrelation of the first-order error term was not rejected. Therefore, it could be finally determined that there was no first-order autocorrelation of the error term.

## Discussion

### Theoretical Implications

Our results suggest extensions to the present literature in two key areas: (1) methodology and (2) organizational adaptation. First, we discovered the possibility of alternative analysis tools using algorithms that went beyond the fundamental limitations of the properties of questionnaire data and financial data. In this study, the panel model was processed by constructing a dictionary and developing algorithms, extracting time series scores, and using them in an empirical analysis, rather than analyzing existing survey or financial data. The data obtained from the survey is “cognitive” data of respondents (Groves et al., 2009), accompanied by response error according to adaptive response behavior (Baumgartner and Steenkamp, 2001; Drolet and Morrison, 2001), and the common method bias (Podsakoff, 2003; Conway and Lance, 2010), which occurs when measuring independent and dependent variables simultaneously. In addition, previous studies contain unresolved problems such as questionnaire order according to context effect (Schuman et al., 1981), problems of symbolism and expression of questions (Fee, 1981), and a non-response problem in which information was not obtained from part of the sample (Hedderley and Wakeling, 1995; Dufour et al., 2001; Groves, 2006). In addition, financial data represents short-term figures and is not free from endogenous problems with performance indicators. On the other hand, DC data extracted from unstructured text through algorithms is primary data that directly analyzes the contents of a company’s ESG management strategy. Therefore, it can be said that the rigor of the data value is relatively high. Moreover, it can be said that this study contributes to academic and methodological advancement in the field in the sense that time series data capable of panel model analysis was extracted.

Measurement of ESG management, which is a non-financial performance, is essentially difficult to quantitatively evaluate, making it a proxy variable as an indicator evaluated by external entities such as the internationally validated MSCI ESG index (total of 7 stages including AAA, AA, A, BBB, BB, B, CCC), Europe’s FTSE4Good index, and latecomer, DJSI index. In the case of Korea, the ESG grades of the Korea Corporate Governance Service (A+, A, B+, B, below B, C, D, etc.), which are calculated on a yearly basis, are used as an authoritative data. These indices take the form of ranking-order variables that assign value according to the evaluation results, so it is necessary to convert them into scores. The problem is that all of them address incompleteness when time series data are needed because this data is not continuous.

Secondly, we explored how DCs interact with environmental uncertainty in causing changes in performance levels. Wu’s (2010) study examined the effects of integrating, learning, and reconfiguring capabilities on individual competitive advantages such as market response speed, production efficiency, product quality, and innovation speed, but rather, detailed consideration of the process of DCs interacting with uncertainty is rare. In this study, DCs were divided into 10 detailed activities (Teece, 2007) and the process of their interacting with uncertainty was examined. Unlike static approaches shown by previous studies on DCs, this study tells us about the importance of the sequence and fit of sensing-seizing-reconfiguring of corporate strategies, and it presents important empirical insights into the dynamic process in which the structural relationship between DCs, competitive advantages, and corporate performance is moderated by uncertainty.

Only 5 out of 10 constructive capabilities of DCs were found to have a statistically significant positive or negative effect on corporate performance. This is partially inconsistent with previous studies (Wu, 2006; Vanpoucke et al., 2014; Kuo and Tsou, 2017; Jiao et al., 2019) that discovered the positive effect of DCs on corporate performance or competitive advantages in general. According to the results of this study the process in which DCs affect a company’s strategic choices and core competitive advantages is not automatic (Ambrosini and Bowman, 2009). In the process of organizational adaptation to ESG management, unlike a previous study (Helfat, 1997) that showed DCs have a positive effect on corporate performance, no automation was found. Rather, there are subordinate activities (capabilities) that do not directly affect corporate performance. In addition, it can be observed that the subordinate activities (capabilities) of DCs with positive or negative direct effects on corporate performance were moderated, such as the disappearance of the direct effect or the change in the direction of the correlation coefficient due to the moderating function of uncertainty. Therefore, it is attractive for strategic management researchers to open the black box using an advanced research model based on DCs moderated by uncertainty.

Third, the existence of uncertainty reminds us that ownership-specific advantages that constitute corporate heterogeneity gradually accumulate and have temporary properties. In the academy of strategic management, it is understood that a company is a bundle of technology and knowledge, and its process of accumulating competitive advantages is gradual rather than radical. Typically, Teece et al. (1997) understand that the essential elements of competitive advantages are accumulated through a gradual path dependence mechanism. In the same vein, Prahalad and Hamel (1994) and Hamel and Prahalad (1994) describe the basis of a company’s core competencies as an evolutionary outcome of its corporate experience.

Gradually accumulated competitive advantages are established as “sustainable competitive advantages” by meeting VRIN (Value, Rareness, Imitability, Non-substitutable) conditions (Barney, 1991). Interestingly, however, Wiggins and Ruefli (2002) revealed that only a few companies have sustainable competitive advantages. Rather, competitive advantage, which is valuable in seizing market opportunities and avoiding threats in a business environment with high uncertainty, should be continuously transformed in accordance with technological and market changes, rather than having sustainability as an attribute. Wiggins and Ruefli (2002) also revealed that over time, the period for companies to maintain their competitive advantages becomes shorter. In the same context, the core competencies presented from the static RBV do not create sustainable competitive advantages in a contemporary era with high environmental uncertainty. On the other hand, DCs do not simply strengthen corporate competitive advantages, but induce them to improve corporate performance by converting them to suit environmental changes.

### Managerial Implications

In addition to the implications for theoretical development in the fields of international business and strategic management, the results of this study provide the following managerial implications for corporate managers. First, it is necessary to discover the source of competitiveness both inside and outside the company as part of the fundamental response strategy to be taken by companies in the era of rising uncertainty. Corporate competitive advantages can be defined using a variety of concepts, including product quality, differentiation ability, cost leadership, flexible process, established customer service, innovation speed, market response speed, production efficiency, cost efficiency, and product line width and depth (Morrison and Roth, 1993; Antonio et al., 2007; Kristal et al., 2010; Wu, 2010). DCs are necessary factors to secure and maintain these competitive advantages (Teece, 2007; Kuo et al., 2017), and to strengthen competitive advantages in response to environmental changes by integrating corporate resources and improving asset utilization (O’Reilly<suffix>III</suffix>, and Tushman, 2008; Li and Liu, 2014; Kuo et al., 2017).

The problem is that the more dynamic industries experience market changes, i.e., changes in consumer needs due to rapid technological changes, the more difficult it is to survive in the market with only the company’s internal technology and knowledge base. Developing new products in line with changes in the business environment requires complementary capabilities of external companies based on internal technologies and knowledge capabilities. As a result, open innovation through strategic alliances or joint ventures is emerging as an attractive option.

Second, it is necessary to maintain strategic value through knowledge-based reconfiguration. In a situation where the contemporary business environment is rapidly changing, it is difficult to maintain a knowledge base as a sustainable competitive advantage (Ambrosini and Bowman, 2009). Under uncertainty, companies must continue to reconfigure to meet detected technology and market changes in order to keep their knowledge base up to date and maintain its strategic value (Leonard-Barton, 1992; Wohlgemuth and Wenzel, 2016).

In this process, it is very important to maintain the fit of sensing-seizing-reconfiguring capabilities. From the fact that the number of firms that recovered after losing their competitive advantages has increased, Wiggins and Ruefli (2002) concluded that successful firms do not retain long-term competitive advantages, but rather create chains of temporary competitive advantages. In the same vein, D’Aveni and Gunther (1994) also stress the importance of realizing a temporary chain of competitive advantages in the era of hyper competition, where the durations of corporate competitive advantages have been shortening. This chain of competitive advantages cannot be realized simply by strengthening DCs. Rather, it is important to strategically appropriate the structural relationship between sensing-seizing-reconfiguring capabilities that constitute DCs so that they can seize opportunities according to market changes. The disconnection between sensing-seizing-reconfiguring capabilities hinders DCs from becoming sustainable competitive advantage resources.

Third, it is necessary to be wary of the strategic failures of DCs caused by the disproportionate pursuit of sensing-seizing-reconfiguring capabilities in ESG management. Unlike CSR activities that emphasize corporate citizenship, ESG is a concept of sustainable investment linked to investor indicators consisting of three specific factors: environment, society, and governance. The emphasis on ESG management has begun to take hold in Korea more by private investors rather than in the public sector. Representatively, Goldman Sachs and JP Morgan, along with BlackRock, the world’s largest asset management company, have expressed their intention to exercise their voting rights against companies that are passive in publicizing their statements of ESG management. In particular, the financial sector responds faster than other industries. There is a consensus that ESG management has a positive effect on the corporate value of financial institutions (Carroll and Einwiller, 2014; Goloshchapova et al., 2019), and it drives a corporation’s financial performance (Friede et al., 2015; Mozaffar et al., 2017; Henisz et al., 2019). On the other hand, there is also a conflicting view that it is not easy to drive financial performance because of the nature of ESG management, which involves many responsibilities resulting in costs (Robert and George, 2013).

According to the results of this study, the DCs embedded in ESG management have a higher sensitivity to market performance than financial performance. The sensitivity of sensing-seizing-reconfiguring is observed in both financial and market performance, but the interaction effect with uncertainty is much more sensitive to market performance. In addition, while the direct effect of sensing-seizing-reconfiguring capabilities have both positive and negative effects, in the case of financial performance, sensing-seizing-reconfiguring capabilities do not have a significant effect due to the moderating effect of uncertainty. On the other hand, in the case of market performance, sensing-seizing-reconfiguring capabilities negatively or positively affect performance based on the moderator, i.e., uncertainty. However, since a significant influence is observed only in the sensing and reconfiguring activities, imbalanced pursuit between the sensing-seizing-reconfiguring capabilities is observed in the case of market performance.

Given these differences in the effects of DCs embedded in ESG management on financial and market performance, it is possible to conclude that the results of the strategic move to ESG management are closer to failure than success in terms of corporate organizational adaptation to environmental uncertainty. As a result, companies should successfully pursue business model transformation to seize market opportunities in the process of sensing market changes in terms of socially responsible investment value and reconfiguring resources and capabilities, rather than establishing a vision and mission from a traditional CSR perspective.

### Limitations and Future Research

Although acknowledging its academic and practical contribution to the academy of strategic management by presenting an alternative analysis tool to increase the objectivity of analysis and opening a black box of DCs embedded in ESG management, this study does not provide us with a full understanding of DCs. Our understanding of DCs can only become more complete when encompassing several future research topics. First, it is necessary to consider the curvilinear effect, not the linear effect of uncertainty. In this study, positive and negative functions were considered, omitting the curvilinear relationship between DCs and uncertainty. Second, further research is needed to examine the interaction effect with uncertainty in specific value chain activities in each of the three areas of DCs, i.e., sensing-seizing-reconfiguring. Third, in future studies, selected companies should belong to a meaningful industry because that industry will be carrying out significant activities which need consideration in the field of DCs. Thirdly, it is also necessary to compare the theoretical and practical meanings of different conceptualizations of DCs presented by Teece (2007); Eisenhardt and Martin (2000), and Zollo and Winter (2002), which are well known for their definition of DCS. Finally, in order to extend the generalizability of this study for the theorizing of DCs, comparative research needs to be done across various industries.

## Data Availability Statement

The raw data supporting the conclusions of this article will be made available by the authors, without undue reservation.

## Author Contributions

BY designed the research method, collected and analyzed the data, and wrote the manuscript. OY was responsible for the conceptualization of the idea and constructed the fundamental theory. Both authors contributed to this article and approved the submitted version.

## Conflict of Interest

The authors declare that the research was conducted in the absence of any commercial or financial relationships that could be construed as a potential conflict of interest.

## Publisher’s Note

All claims expressed in this article are solely those of the authors and do not necessarily represent those of their affiliated organizations, or those of the publisher, the editors and the reviewers. Any product that may be evaluated in this article, or claim that may be made by its manufacturer, is not guaranteed or endorsed by the publisher.
